# Seroepidemiology for Enteric Fever: Emerging Approaches and Opportunities

**DOI:** 10.1093/ofid/ofad021

**Published:** 2023-06-02

**Authors:** Kristen Aiemjoy, Jessica C Seidman, Richelle C Charles, Jason R Andrews

**Affiliations:** Division of Epidemiology, Department of Public Health Sciences, University of California, Davis School of Medicine, Davis, California, USA; Department of Microbiology and Immunology, Faculty of Tropical Medicine, Mahidol University School of Medicine, Bangkok, Thailand; Sabin Vaccine Institute, Washington, District of Columbia, USA; Division of Infectious Diseases, Massachusetts General Hospital, Boston, Massachusetts, USA; Department of Medicine, Harvard Medical School, Boston, Massachusetts, USA; Department of Immunology and Infectious Diseases, Harvard T. H. Chan School of Public Health, Boston, Massachusetts, USA; Division of Infectious Diseases and Geographic Medicine, Stanford University School of Medicine, Stanford, California, USA

**Keywords:** disease burden, enteric fever, seroepidemiology, serosurveillance, typhoid

## Abstract

Safe and effective typhoid conjugate vaccines (TCVs) are available, but many countries lack the high-resolution data needed to prioritize TCV introduction to the highest-risk communities. Here we discuss seroepidemiology—an approach using antibody response data to characterize infection burden—as a potential tool to fill this data gap. Serologic tests for typhoid have existed for over a hundred years, but only recently were antigens identified that were sensitive and specific enough to use as epidemiologic markers. These antigens, coupled with new methodological developments, permit estimating seroincidence—the rate at which new infections occur in a population—from cross-sectional serosurveys. These new tools open up many possible applications for enteric fever seroepidemiology, including generating high-resolution surveillance data, monitoring vaccine impact, and integrating with other serosurveillance initiatives. Challenges remain, including distinguishing *Salmonella* Typhi from *Salmonella* Paratyphi infections and accounting for reinfections. Enteric fever seroepidemiology can be conducted at a fraction of the cost, time, and sample size of surveillance blood culture studies and may enable more efficient and scalable surveillance for this important infectious disease.

The incidence of enteric fever, an invasive bacterial infection caused by *Salmonella enterica* serovars Typhi and Paratyphi, is largely unknown in regions without blood culture surveillance [1]. Safe, effective vaccines against *S.* Typhi are available and are recommended for use in high-burden areas by the World Health Organization. However, many countries lack the reliable, high-resolution data on enteric fever burden needed to decide when and among whom to introduce the vaccine. Moreover, without data to demonstrate burden, countries may face difficulties accessing funds offered by Gavi, the Vaccine Alliance to support typhoid conjugate vaccine (TCV) introduction. This paradox fuels deepening inequities in disease occurrence, whereby countries without robust surveillance systems cannot generate the data needed to make informed decisions about whether and how to prioritize TCV introduction amid other vaccine investments.

Enteric fever incidence is often estimated using longitudinal blood culture surveillance studies that count the number of culture-confirmed enteric fever patients reporting to select health facilities and, where possible, making adjustments for healthcare-seeking patterns [2, 3]. Blood culture is highly specific but suffers from several shortcomings, in that it requires sufficient laboratory capacity, has moderate sensitivity, which may be further diminished by antibiotic use, and is relatively costly to perform [4, 5]. As a result, blood culture is only available in a limited number of settings, often more well-resourced urban centers. Reliance on this resource-intensive method of estimating incidence means that data from a small number of studies are often extrapolated to cover large, heterogenous areas within and across countries, leaving significant gaps in our understanding of the actual underlying burden of enteric fever in many at-risk communities [1, 3].

Seroepidemiology, an approach using immunological markers of pathogen exposure combined with population sampling strategies, has the potential to greatly expand the geographic scope of typhoid surveillance to target areas with limited or no access to blood culture [6]. Here, we review historical and emerging literature on typhoid seroepidemiology and discuss opportunities and challenges for its implementation.

## SEROLOGIC MARKERS FOR TYPHOID DETECTION

Research on serologic markers for enteric fever has primarily focused on their diagnostic utility. The Widal test, developed by French physician-microbiologist George Widal in 1896, measures serum agglutinating antibodies to *S.* Typhi's flagellar H and somatic O (lipopolysaccharide [LPS]) antigens [7]. This assay remains widely used globally despite ample evidence of poor performance, including modest sensitivity and specificity, with variable predictive value across geographic areas [8–10]. Several alternative antibody-based rapid diagnostic tests have been developed over the past century, including TUBEX and Test-it Typhoid, which measure antibodies against *S.* Typhi LPS, and Typhidot, which measures antibodies to a 50 kDa outer membrane protein. Few tests have surpassed 90% sensitivity and/or specificity, with none meeting recently suggested target product profile benchmarks [11].

As for markers of infection transmission in populations, most studies have measured immunoglobulin G (IgG) responses for the Vi capsular antigen of *S.* Typhi. Vi IgG has been used as a seroepidemiologic marker since the 1980s across many settings, including South Africa, Nepal, Fiji, Bangladesh, and Malawi [12–15]. However, Vi IgG responses may not reliably increase following infection. For example, a recent longitudinal study in Nepal showed that only a quarter of blood culture–confirmed cases had elevated anti-Vi IgG responses after infection [16]. Additionally, in settings where seropositivity and clinical incidence data have been compared, a clear association has not been identified [12, 13, 15]. In Fiji, age-dependent Vi responses were very similar across communities with markedly different clinical incidence rates [12]. A further limitation is that the typhoid conjugate vaccine induces a Vi IgG response [17]. As Vi seroresponse cannot distinguish between vaccination and natural infection, it may not be useful for estimating disease burden in settings where Vi-based vaccines are introduced.

*Salmonella* Typhi hemolysin E (HlyE) has been identified in two independent studies as a promising marker to identify *S.* Typhi and Paratyphi infections [18–21]. HlyE is a pore-forming toxin found in *S.* Typhi and Paratyphi but rarely seen in other *Salmonella* species [22]. Anti-HlyE immunoglobulin A (IgA) antibodies, alone or in combination with anti-LPS IgA antibodies, were found to have high sensitivity and specificity (>90%) to distinguish acute typhoid infection from other invasive bacteremias in febrile patients in Bangladesh and Nepal; these same antigens were significantly elevated in Bangladeshi typhoid cases compared to healthy controls [18]. A study of enteric fever in Malawi using blood culture and polymerase chain reaction (PCR) to identify invasive non-typhoidal *Salmonella* (iNTS) and *S.* Typhi–positive patients found that anti-HlyE IgG and immunoglobulin M antibody levels were significantly elevated over controls who were febrile and typhoid negative by blood culture and PCR and over afebrile, healthy controls [23]. In a recent multisite study, HlyE was shown to be an accurate marker of enteric fever transmission in populations [16] and is discussed in more detail in the following section.

## ANALYTICAL APPROACHES FOR TYPHOID SEROEPIDEMIOLOGY DATA

Seroepidemiology is an approach to estimating disease burden where antibody levels are measured in populations regardless of symptomatic illness to characterize pathogen exposure. Unlike molecular- and microscopy-based diagnostics, which detect active infections, quantitative antibody levels offer a window into the history of exposure. Two important seroepidemiology metrics to characterize disease burden are seroincidence and seroprevalence. Seroprevalence describes the percentage of the sampled populations that is seropositive, whereas seroincidence describes the rate at which new infections occur in a population.

Defining a cutoff to classify a population into two groups (seropositive and seronegative) is often difficult, as the distribution of antibody responses in many populations is not bimodal, and reference standard–based positivity thresholds have not been identified [24]. Cutoffs are further complicated by the waning of antibody responses and variation of antibody responses by age, both of which exist for responses to typhoidal *Salmonella* antigens HlyE and *S.* Typhi LPS. When antibody responses do not wane or wane very slowly—like Vi IgG—the seroprevalence will be a function of the age structure of the sampled population. In such circumstances, estimating seroincidence can be difficult as there will be a saturation effect at young ages in populations with high transmission intensity.

**Figure 1.**
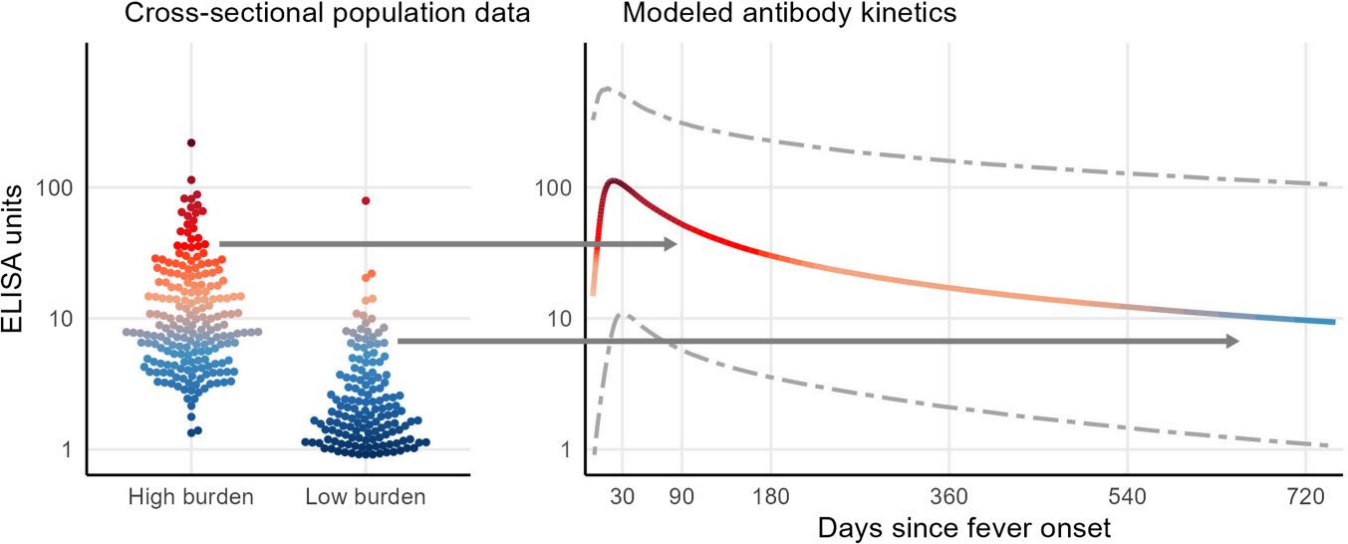
Conceptual diagram for cross-sectional seroincidence estimation. The left panel illustrates quantitative antibody responses from a cross-sectional serosurvey conducted in both high and low burden settings. Each point corresponds to an individual's antibody response, measured in kinetic ELISA units, with the y-axis presented on a log scale. The right panel demonstrates antibody dynamics following confirmed enteric fever infection. The solid line represents the median response, while the dashed lines correspond to the upper 97.5th percentile and lower 2.5th percentile. The antibody kinetics are used to infer the most likely exposure time for each individual point in the cross-sectional serosurvey (as shown in the left panel), while accommodating heterogeneity in antibody responses.

New methods are available to estimate enteric fever seroincidence using information about the antibody decay kinetics among confirmed cases [16, 25]. The peak response and decay rate act like a clock, allowing inference about where an individual sample falls on the decay curve and when they were most likely exposed (Figure 1) [26–28]. At the population level, it is possible to calculate seroincidence as the rate most likely to result in the observed cross-sectional quantitative antibody responses. In high-incidence settings, more individuals will have been recently infected and have high antibody concentrations, compared with a low-incidence setting. Unlike classic methods, which ignore individual-level heterogeneity in antibody responses, heterogeneity is modeled with this approach and explicitly incorporated into the uncertainty intervals around the seroincidence estimates. Additional advantages of this method are that it accommodates 2 additional sources of uncertainty common in serologic response data: measurement error (repeatability) and nonspecific binding [25]. Moreover, leveraging information about decay rates makes it possible to characterize incidence in settings with very high transmission where dichotomized IgG responses would be saturated.

The recent Seroepidemiology and Environmental Surveillance study (SEES) validated this method in a multisite study in Bangladesh, Nepal, Pakistan, and Ghana. Longitudinal anti-HlyE and LPS antibody responses were measured among blood culture–confirmed enteric fever cases and the antibody kinetics were modeled. The antibody kinetic models were then used to calculate the population-level seroincidence rate. The serosurveys were conducted in the same catchment areas of ongoing prospective blood-culture surveillance studies, allowing for the comparison of findings across the surveillance strategies [16].

In acute cases, the anti-HlyE and LPS antibodies (IgG and IgA) peaked in the first 21 days following fever onset, decayed over the duration of follow-up, and were significantly elevated over the average level observed in the population surveys. The longitudinal antibody kinetics were similar across countries, showing a slower IgG decay rate in higher-burden communities. Using the antibody decay parameters, the study found seroincidence rates ranging from 6000 to 59 000 infections per 100 000 person-years, significantly higher than clinical incidence rates. The rank order of seroincidence rates among young children across the 5 sites was the same as the rank order of clinical incidence rates, with the resolution to distinguish between locations across a range of clinical incidence rates. These findings suggest this approach can rapidly and accurately estimate typhoid seroincidence from cross-sectional serosurveys.

## OPPORTUNITIES FOR ENTERIC FEVER SEROEPIDEMIOLOGY

A leading opportunity for enteric fever seroepidemiology is to expand surveillance to settings that lack blood culture–based surveillance capacity. This information is critical to public health policymakers as they decide where and among whom to prioritize vaccine introduction. Serosurveillance for enteric fever presents notable logistical and design advantages relative to blood culture surveillance. From the logistical perspective, a serosurvey can be conducted for a lower cost and over a much shorter timeframe than a longitudinal cohort study. For example, the recent SEES studies demonstrated that a population-based sample could be performed in a community over a period of weeks to months, achieving greater precision than a clinical incidence study conducted over 2 to 3 years without compromising accuracy [16]. Another logistical advantage is that the serologic assays discussed above require just a small volume of blood and can be implemented using capillary blood collected onto filter papers (also known as dried blood spots). Dried blood spots do not require the extensive cold chain required for collecting venous blood, yielding additional cost and efficiency benefits. Moreover, because collecting dried blood spots is minimally invasive, participation may be higher, and there are fewer opportunities for selection bias, especially for young children.

**Figure 2.**
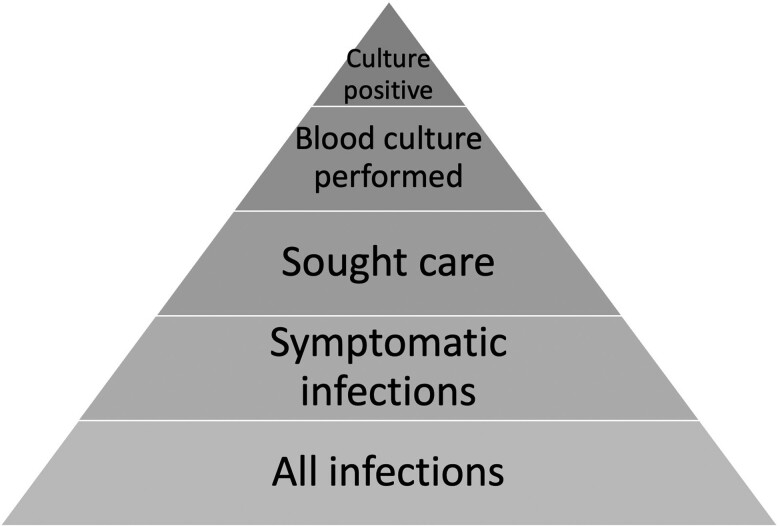
Enteric fever surveillance capture pyramid, where the base represents all infections and the apex represents culture-positive cases detected at surveillance sites.

In addition to logistical advantages, estimating enteric fever incidence from population-based serosurveys has several positive study design attributes. First, household-based serosurveys are unbiased by healthcare-seeking patterns, which vary considerably by key sociodemographic variables (eg, wealth) that also predict typhoid exposure risk and can result in biases in estimates obtained from hospital-based studies. Second, serological assays are less likely to be affected by antibiotic use during illness, which compromises the sensitivity of blood cultures. Third, seroincidence captures subclinical and mild infections, which are unmeasurable using conventional blood culture surveillance systems. Thus, estimates based on serology do not need to be adjusted for care-seeking or the sensitivity of blood culture (Figure 2). Because seroconversions are a more frequent outcome than blood culture–confirmed disease, incidence can be estimated with greater precision on much smaller sample sizes. For example, the SEES study found that sample sizes of 200 to 400 individuals per age strata were sufficient to reliably estimate seroincidence in settings ranging from high to low disease burden [16].

Beyond surveillance, seroepidemiology’s logistical and design advantages may be valuable for monitoring vaccine impact. Typhoid vaccine efficacy studies are powered to detect differences in blood culture–confirmed cases. Recent randomized trials have enrolled >20 000 participants to detect <80 cases of typhoid fever [29, 30]. As discussed above, these cases are a small fraction of the underlying typhoid burden. With serologic outcomes, there is greater statistical power to detect differences; therefore, smaller sample sizes are required. Further, cluster-randomized vaccine trials are often not powered to detect indirect effects, which involve comparing outcomes among a relatively small number of unvaccinated individuals. Because seroincidence is higher than clinical incidence and can be measured at a single time point, studies employing seroepidemiology may have greater power to detect indirect effects and other subgroup outcomes that may not be possible when using clinical endpoints.

Recent advances in bead-based multiplex assays have created an opportunity for multipathogen serosurveillance [31]. This technology can simultaneously evaluate immune responses to multiple antigens with minimal sample volume. This integrated serosurveillance approach is increasingly emerging as an efficient means for population-based surveillance of vaccination coverage and infectious diseases of public health importance. Enteric fever serologic markers can be incorporated into existing or future multiplex-bead assay panels and will expand opportunities to obtain burden estimates for enteric fever once validated.

Last, an efficient approach to generate enteric fever seroincidence estimates is to test previously collected and banked blood samples. Serosurveys are becoming increasingly common and often collect more blood than is required for the primary outcome. These banked samples present an opportunity to rapidly generate enteric fever seroincidence estimates. This approach was recently employed using banked dried blood spots collected for a severe acute respiratory syndrome coronavirus 2 serosurvey in Juba, South Sudan, identifying a high and previously unrecognized burden of enteric fever there [32].

## CHALLENGES AND FUTURE DIRECTIONS

Methods for enteric fever seroepidemiology are still evolving, and as such, there remain challenges and limitations. First, there are currently no antigens available that can reliably distinguish *S.* Typhi from *S.* Paratyphi A serovars, which account for around a quarter of all enteric fever cases globally [1]. As *S.* Typhi–specific vaccine use grows, characterizing the population-level burden of *S.* Paratyphi A versus *S.* Typhi will become increasingly relevant. Anti-HlyE responses are observed in both *S.* Typhi and *S.* Paratyphi A infections, and therefore, using anti-HlyE responses as a serologic marker for overall enteric fever may bias estimates of vaccine efficacy toward the null.

Next, seasonality in infection risk may influence estimates if not accounted for in the study design or analysis. We know that enteric fever incidence is seasonal in endemic areas and that transmission intensity is often variable from year to year. Therefore, the season when a serosurvey is conducted will influence the disease burden estimated in that population. For example, a serosurvey conducted just after these surges in disease incidence will show a higher burden than one conducted during low-transmission calendar periods. Currently, the best way to handle seasonality is to enroll individuals consistently over a year or to conduct repeated cross-sectional surveys in the same area in high- and low-transmission seasons. Future methodological work is needed to adjust seroincidence and seroprevalence estimates to account for seasonal variation in infection risk.

Another challenge for interpreting serologic responses for enteric fever is characterizing and incorporating information about reexposures. In regions with high transmission intensity and in higher-risk populations, individuals may be frequently reexposed to enteric fever. The quantitative peak antibody response and the shape and rate of antibody decay will likely differ among reinfections compared to primary infections. All cutoff-based analytic approaches ignore this dimension. In the SEES study, the authors chose to identify and remove likely reinfections from the antibody kinetic modeling. They assumed that dynamics were similar among primary and reinfections for the seroincidence modeling [16]. Future work is needed to characterize antibody dynamics after reexposures and incorporate this information into describing population-level disease burden. The impact of prior vaccination on antibody dynamics will also need to be evaluated.

Finally, seroepidemiology captures both symptomatic and subclinical infections, unlike classic epidemiologic study designs, which typically focus on clinical disease. Characterizing clinical disease is important for quantifying attributable morbidity and mortality in a region. It is unknown by what magnitude seroincidence exceeds clinical incidence or if such a calculation can be made and extrapolated across sites with varying degrees of access to blood culture diagnostic facilities. Yet, given that we know care-seeking behaviors strongly influence clinical disease surveillance, population-level seroincidence provides a less biased description of enteric fever transmission in a population.

## CONCLUSIONS

Enteric fever remains a significant cause of morbidity and mortality globally. Many of the most at-risk countries lack adequate blood culture surveillance systems needed to characterize disease burden, particularly outside major urban areas. These missing data are critical to qualify for financial subsidies to support vaccine introduction and understand where and among whom to prioritize public health interventions. Addressing surveillance disparities may, in turn, improve equity in access to vaccination and other interventions by directing resources toward communities at greatest need rather than those with the best surveillance infrastructure. Enteric fever seroepidemiology can be conducted at a fraction of the cost, time, and sample size of blood culture surveillance studies and warrants inclusion in the library of tools urgently needed to characterize and reduce the burden of enteric fever globally.

## Notes

***Financial support.*** The Bill & Melinda Gates Foundation; NIH NIAID (K01 TW012177).

***Supplement sponsorship.*** This article appears as part of the supplement “Charting the Course to Meet the Challenges Ahead: Research and Developments on Typhoid and Other Invasive Salmonelloses” sponsored by the Coalition against Typhoid Secretariat, housed at the Sabin Vaccine Institute in Washington, DC and made possible by a grant from the Bill & Melinda Gates Foundation.
